# Electrode materials for stretchable triboelectric nanogenerator in wearable electronics

**DOI:** 10.1039/d2ra01088g

**Published:** 2022-04-07

**Authors:** Irthasa Aazem, Dhanu Treasa Mathew, Sithara Radhakrishnan, K. V. Vijoy, Honey John, Daniel M. Mulvihill, Suresh C. Pillai

**Affiliations:** Nanotechnology and Bio-Engineering Research Group, Department of Environmental Science, Atlantic Technological University, ATU Sligo Ash Lane, Sligo F91 YW50 Ireland pillai.suresh@itsligo.ie; Department of Polymer Science and Rubber Technology, Cochin University of Science and Technology Kerala 682022 India; Inter University Centre for Nanomaterials and Devices, Cochin University of Science and Technology Kerala 682022 India; Materials and Manufacturing Research Group, James Watt School of Engineering, University of Glasgow Glasgow G12 8QQ UK; International School of Photonics, Cochin University of Science and Technology Kerala 682022 India; Health and Biomedical (HEAL) Strategic Research Centre, Atlantic Technological University, ATU Sligo Ash Lane Sligo F91 YW50 Ireland

## Abstract

Stretchable Triboelectric Nanogenerators (TENGs) for wearable electronics are in significant demand in the area of self-powered energy harvesting and storage devices. Designing a suitable electrode is one of the major challenges in developing a fully wearable TENG device and requires research aimed at exploring new materials and methods to develop stretchable electrodes. This review article is dedicated to presenting recent developments in exploring new materials for flexible TENGs with special emphasis on electrode components for wearable devices. In addition, materials that can potentially deliver properties such as transparency, self-healability and water-resistance are also reviewed. Inherently stretchable materials and a combination of soft and rigid materials including polymers and their composites, inorganic and ceramic materials, 2D materials and carbonaceous nanomaterials are also addressed. Additionally, various fabrication strategies and geometrical patterning techniques employed for designing highly stretchable electrodes for wearable TENG devices are also explored. The challenges reflected in the present approaches as well as feasible suggestions for future advancements are discussed.

## Abstract 2

Schematic illustration of the general requirements of components of a wearable TENG.

## Graphical abstract

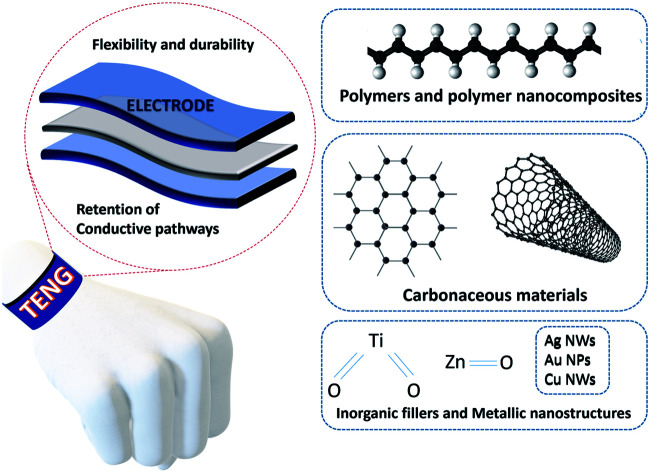

## 1. Introduction

**Fig. 1:**
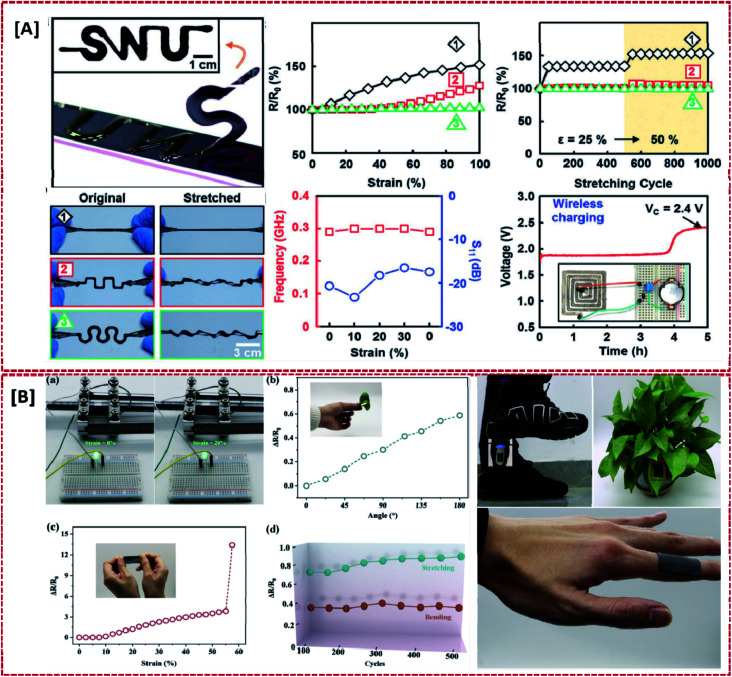
Significance of stretchable electrode TENGs for wearable devices: [A] 100% stretched and un-stretched forms of a patterned CNT electrode showing their durability (reprinted with permission,^73^ copyright 2017 Advanced Functional Materials). [B] Electrical performance and wearability of a PU–GR@MWCNTs stretchable electrode under different conditions of deformation (reprinted with permission,^6^ copyright 2019 Nano Energy).

Harvesting the abundant energy available around us in various forms has been one of the key areas of interest in the global research community. The development of energy-efficient devices to capture and utilize it effectively has become essential. Additionally, the emerging threat of energy scarcity in the modern era also forecasts the relevance of energy capturing and conversion devices in the future. Over the past few decades, various technologies such as piezoelectric, photovoltaic techniques that are capable of harvesting electrical energy from various other forms of energy had been introduced.^1,2^ Recent research have been focusing on developing technologies for generating electricity from mechanical energy which was one of the most dispensed and under-utilized energy forms. Moreover, these technologies find their application in self-powered devices, which has also gained huge demand in modern society. Additionally, recent advancements have made possible low power consumption in portable and self-powered devices. Moreover, the recent worldwide acceptance received by compact, wearable and portable electronics in terms of utility, aesthetics and reliability is an indication of the technological prospect of mechanical energy harvesting devices.^3^ Most importantly, the possibilities offered by such devices in effectively utilizing the otherwise wasted energy in our surroundings has justified the continuing search for new roots of green and sustainable energy. The concept of scavenging mechanical energy from motions occurring in nature and from day-to-day human body movements have been extensively utilized by self-powered sensors and wearable electronic devices. Among them, triboelectric nanogenerator (TENG) based devices which operate by a combination of electrification through contact or friction and electrostatic induction has gained much attention. TENGs had been an emerging area of research, ever since being introduced by Wang *et al.* in 2012.^4^ The capability of TENGs to harness mechanical energy from a wide range of energy sources ranging from minute human body motions to massive ocean waves had offered huge possibilities for exploiting the neglected energy.^5^ Apart from that, high power density, simple and low-priced designing strategies, scalability and integrity with other energy harvesting approaches *etc.*, have marked the uniqueness of TENGs compared to piezoelectric systems, solar energy, electromagnetic induction *etc.*^6^ In a typical TENG, a charge transfer occurs between two surfaces of opposite tribo-polarity when they are in contact with each other. Further, as the layers move apart from each other a potential difference is created which is balanced by the flow of charge carriers between the electrodes attached to the friction. TENGs operate under four different modes which are based on the arrangement of the charge generating layers and the electrodes in the device. They are namely: contact-separation mode, single electrode mode, lateral sliding mode, and free-standing triboelectric-layer mode.^7^ The intervention of next-generation electronics in the present decade has introduced numerous wearable TENG devices and sensors such as electronic skin, flexible and touch screen displays, electronic watches, biomechanical monitoring sensors *etc.*^1,3,8–10^ Consequently, each component of the TENG is expected to be considerably stretchy, flexible, and mechanically stable rendering suitable deformability and wearability. Extensive research has been carried out to make the triboelectric layer, which is responsible for the generation of triboelectric charges in TENG assembly, fully stretchable and reliable for wearable electronics.^8^ At the same time, developing highly stretchable electrodes which is another crucial component of a TENG, is also equally significant for achieving 100% wearability (Fig. 1).

The major role of the electrode in a typical TENG design is to provide the transfer of charges generated in the triboelectric layer to the external circuit with minimum loss. Conventionally, on account of the good electrical conductivity and rigidity offered by them, metallic materials were used to make electrodes for TENGs. In traditional TENG designs, typical metals such as Al, Cu *etc.* were used as the electrode materials because they were only primarily intended to make contact with the triboelectric layer to carry the charge. However, when it comes to wearable electronics, mechanical deformability and flexibility being the primary and most important requirements, rigid materials tend to limit the stretchability and flexibility of the electrode, thereby affecting the wearability of the device.^11^ Moreover, in many of the stretchable TENGs developed recently, the electrode apart from just being a charge carrier to the external circuit acts as one of the tribolayer – the so-called single electrode mode.^12,13^ Thus, exploring stretchable electrode materials for fabricating TENGs with a lower chance of delamination is crucial. Various strategies for fabrication, appropriate geometrical designs and a suitable combination of stretchable, conductive and rigid materials were employed to achieve fully stretchable high-performance electrodes. Multi-functional stretchable electrodes with characteristics such as transparency, self-heal ability, ability to be waterproof, wearability over large area surfaces *etc.* are also achieved through these studies.^5,12,14–17^

Herein, based on recent advancements, we review the various approaches for developing stretchable electrodes for TENGs for wearable electronics. A broader perspective of the desired properties of the stretchable TENGs with special emphasis on flexible electrodes is discussed. Different thermoplastic polymeric materials and their composites with conductive fillers, 2D and carbonaceous nanomaterials and polymer ceramic composites utilized in the recent studies to develop highly stretchable and deformable electrodes are reviewed. Simple and reliable fabrication strategies, geometrical designs and surface patterning are also explored. Future advances and perspectives for the realization of fully stretchable electrodes for wearable applications are also presented.

## 2. General requirements of stretchable components in wearable TENGs

**Fig. 2:**
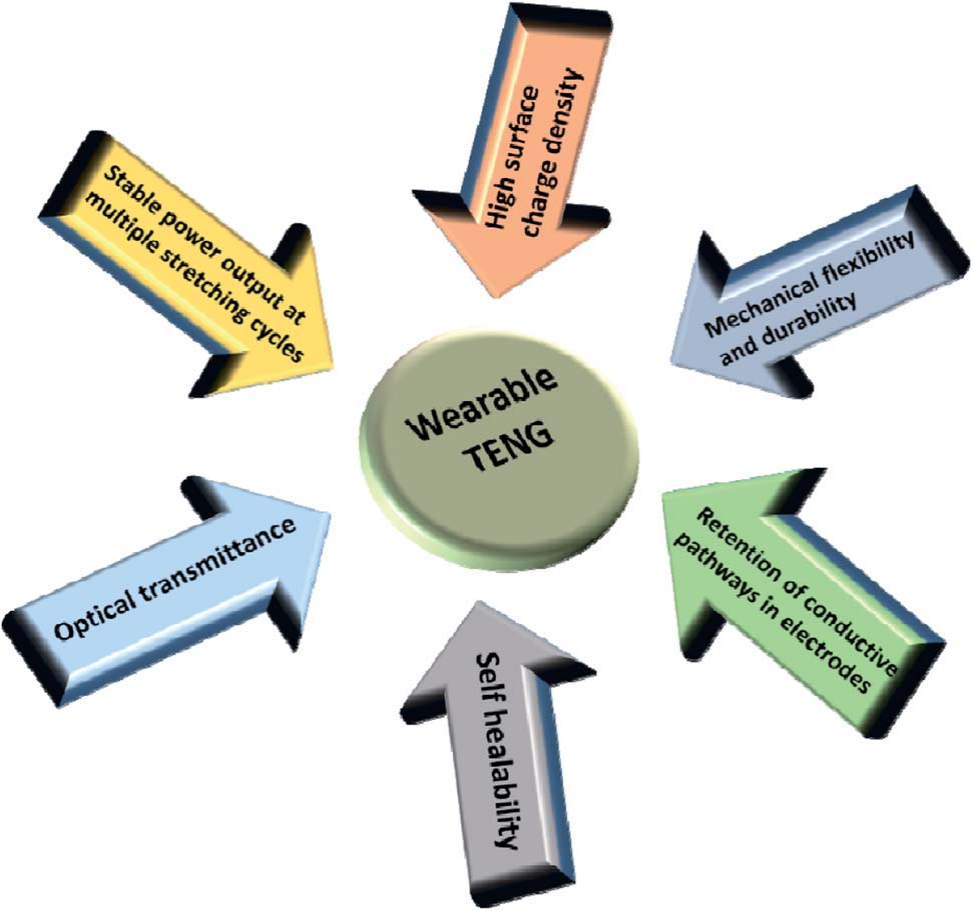
Schematic illustration of the general requirements of components of a wearable TENG.

**Fig. 3:**
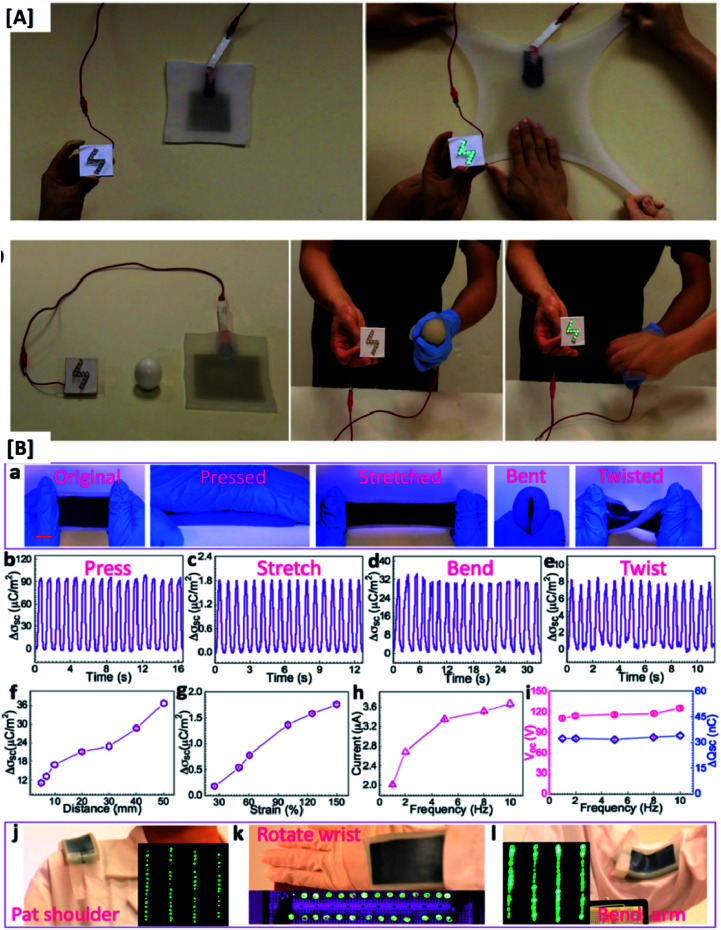
[A] Highly stretchable and mechanically durable electric eel skin inspired TENG and the schematic representation of its energy generating mechanism (reprinted with permission,^11^ copyright 2016 Advanced Materials). [B] Electrical output performance of a flexible and waterproof self-charging TENG under various conditions of deformation (reprinted with permission,^17^ copyright 2016 ACS Nano).

**Fig. 4:**
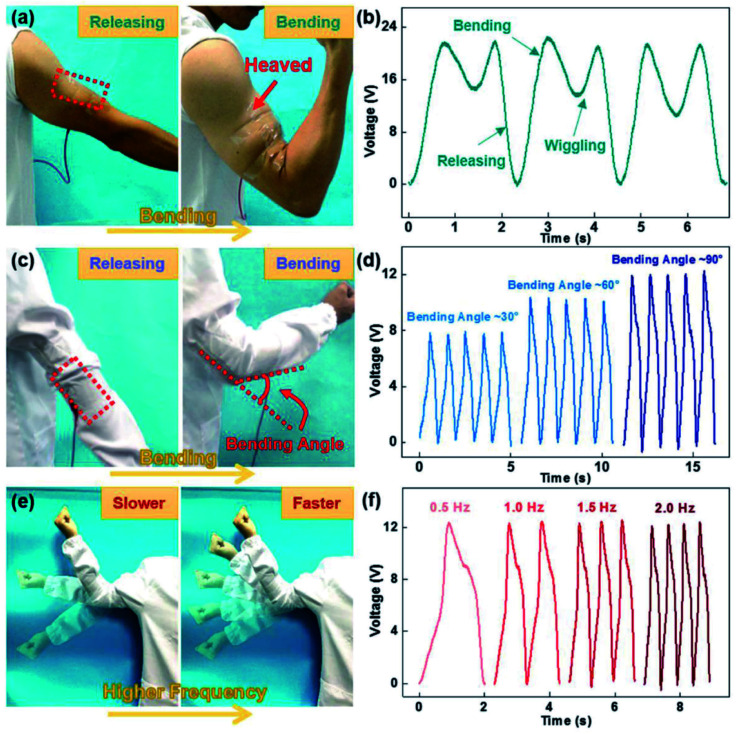
(a–f) Output performance of a highly stretchable wrinkled TENG design based active motion sensor at varying conditions of bending angle and bending frequency (reprinted with permission,^3^ copyright 2018 Advanced Functional Materials).

One of the major requirements associated with TENGs for wearable electronics is their compatibility with irregular or odd surfaces and hostile environments. Apart from that, most of the other basic requirements such as retention of conductive pathways in electrodes, stable power output *etc.* revolves around the stretchability. The common requirements of wearable TENG components are illustrated in Fig. 2. Retaining their output performance under severe conditions of deformation is a challenge in TENGs. As each component of a TENG exhibits variation in terms of its mechanical behaviour and interfacial exchange, the stretchability and flexibility of the device need to be ensured to achieve a fully reliable design. As far as wearable electronics are concerned, the ability of the device to withstand a larger amount of strain is of prime importance, since each component of the TENG would be subjected to frequent unpredictable and complex human body motions such as extension, bending and torsion *etc.* particularly associated with the joints and limbs. Apart from the durability of the device, the output current and voltage shown by these devices are also influenced by the response of these components to the extreme deformations involved, which is clearly demonstrated in Fig. 3 and 4. Consequently, their output performance at varying conditions of mechanical deformations depends upon the response of these components to different strain rates, frequency and load to which the device is subjected to.^6^ This was demonstrated by the electrical output performance shown by a fully stretchable TENG (FSTENG) utilizing a 3D structured porous PDMS layer and a multi-walled carbon nanotube (MWCNT) based electrospun electrode.^6^ A stable performance with an output voltage of 90 V was attained by this electrode and was retained well at a stretching ratio of 50%. This is attributed to the flexibility of both the PDMS friction layer and the polyurethane nanofibers incorporated MWCNT electrodes. An intrinsically mechanically durable skin like TENG (SLTENG) developed by Ying *et al.*^11^ also has demonstrated the influence of mechanical flexibility of a wearable TENG on its output performance, with an efficient power output over 300% strain. The high tear strength exhibited by this silicone rubber-based SL-TENG helped the device to retain its functionalities at extreme deformations with efficient charge transfer. In addition to relying on flexible materials, different types of patterning and geometrical designing could be considered for fabricating the device to make it deformable and stretchable. Various designs like wrinkled surfaces, sandwiched structures, buckling between layers, micro-pyramid patterned surfaces, liquid metal-based shape adaptive designs *etc.* are also utilized in recent studies to achieve mechanical flexibility and stretchability.^3,8,14,18^ Also, providing a flexible outer layer to encapsulate the whole assembly prevents the chances of mechanical ruptures and improves the triboelectric polarizability of the structure. Additionally, in certain cases, the inherent dielectric properties achieved through surface functionalization of the elastic substrates help to avoid the need of incorporating multifunctional materials. Thereby, it simplifies the fabrication without compromising the stretchability good stretchability.

Another important factor required for developing flexible TENGs with better performance is the properties that govern the generation of tribo-charges at the interface. Contacting materials with a significant difference in electron affinity need to be chosen to maximize charge transfer. This is usually made possible by adopting materials from opposite ends of the triboelectric series. Other refinement materials can also be incorporated. For example, ferroelectric materials such as P(VDF–TrFE) can be used to boost TENG output when permanently polarized.^19^ Apart from the inherent material properties, it is also important to achieve a high degree of contact area at the contact interface. This can be achieved in various ways: through ultra-smooth surfaces, *via* conforming surfaces having particular patterns (*e.g.* a hard wavy surface and a soft counter-surface) and by increasing the contact force.^20,21^ For textile TENGs, achieving good levels of the contact area is a difficult challenge and is the subject of ongoing research.

Owing to the general insulating nature of dielectric tribomaterials, the charge transfers from the triboelectrification layer to the electrode (*via* electro-static induction) in TENGs is of great concern. Therefore, proper surface engineering at the electrode surface and friction layer/electrode interface along with the optimization of the right combination of electrodes with appropriate work functions and triboelectric material is required for an increased surface charge density and power output.^22^ At the same time when it comes to developing the electrode for stretchable TENGs, the combination of rigid conductive materials with soft and deformable materials along with appropriate design strategies need to be considered. Incorporating soft polymeric matrices with flexible and conductive fillers and providing an intermediate thin polymeric film with tunable conductivity is a common strategy employed to achieve this. In such cases, different from the conventional rigid metal electrodes, the dielectric properties of the materials could be improved by optimizing the volume fraction of the filler to favour the entrapment of more electrons. This in turn can reduce the possibility of a loss of electric current between the triboelectric film and the electrode without compromising the flexibility of the components. The dielectric polarization of the tribomaterials could remain underutilized if the filler materials happen to trap some charge carriers in the electrode because of remnant polarization shown by certain materials, especially inorganic fillers.^23^ Therefore, proper selection of dielectric materials yielding maximum transfer of charges to the external circuit and optimization of their volume fraction is important.

One of the main concerns with patchable TENGs is the unstable electrical power output due to repetitive and non-uniform stretching cycles. To resolve this, it is highly important to have good conductivity for the electrodes to facilitate an effective and continuous flow of electrical charges to maintain the expected power output. The retention of conductive pathways of stretchable electrodes unaffected during various conditions of stretching is also crucial to maintain the performance of TENGs over many stretching cycles. Here again, stable triboelectric charge generation could be achieved by embedding inherently flexible materials with certain conductive nano fillers of different morphologies. The formation of interconnecting networks between these nanoparticles helps to maintain the integrity of conductive pathways in the electrode and thereby ensure stable output.^18^ Stretchable electrodes with conductive fibre structures are also found to be exhibiting continuous stable power output. For example, a conductive fibre based TENG developed by Jiwon *et al.* featured a fully stretchable electrode with high electrical output. The 100% stretchability, stable electrical output with repetitive deformation cycles and the feasibility of being tailored onto a large surface area were achieved by this design.^24^ The woven structure of this conductive fibre fabricated with several strands helped to achieve a stable output and maximum contact with the human skin. Another example of a highly stretchable TENG with good conductivity is an organogel electrode-based triboelectric fibre demonstrated by Jing *et al*.^25^ A combination of thermoplastic polyurethane (TPU) with carbon black (CB) was observed to yield excellent stretchability and conductivity in the work conducted by Zhang *et al.*^12^ The increase in the amount of CB in TPU matrix improved the conductive pathways of the electrode maintaining the stretchability provided by the TPU domains up to 40% of CB loading. On varying the amount of CB in the TPU matrix, the formation of a percolating conductive network was observed. Consequently, the conductivity of the composite was increased by several orders of magnitude. This highly conductive and flexible electrode eliminates the problem of cracking observed in metallic electrodes and is also highly tailorable and wearable. It also exhibited better output in the stretched state without mechanical damage. This system which possessed a denser interlinked network of CB fillers is also a good example of the ability of flexible thermoplastic elastomers to accommodate a considerable amount of filler without affecting their flexibility.^26^ In all these cases, it can be observed that the stretchability comes from the polymeric domain and does not interfere with the conductive pathways provided by the other component. The major factor to be considered in obtaining excellent surface charge density and power output for TENGs used in wearable application is to render the electrode material highly conductive. The percolation threshold of the conductive entities of the electrode design determines the effectiveness of accumulation of charges without leakage. Apart from that, the interlinkages formed between conductive fillers provide continuous flow of current in the electrode. The orientation and integrity maintained by nanostructured conductive materials at repetitive and extreme stretching cycles is also favorable to maintain the stability of output performance in stretchable TENGs. Additionally, dual network systems in which a major portion of the external force is distributed uniformly over the continuous phase such as polymeric nanofibers are also highly effective in preserving the conductive pathways upon extreme conditions of deformation.^12^ Moreover, the appropriate patterning of the surface to withstand large amount of strain and ensure maximum contact with the operating surface could be considered for achieving good charge transfer.

Additional functionalities such as transparency, self-healability *etc.* are also to be considered while designing wearable TENGs. This calls for the utilization of multifunctional materials and appropriate techniques of simple and adaptive geometrical designs and configurations to impart high flexibility, deformability, and durability of TENGs. Following this, a wide range of materials and simple techniques of fabrication have been investigated recently to develop high-performance wearable devices with attributes such as light weight, flexibility, transparency and self-healing properties. Frequent stretching and release experienced by wearable devices lead to cracking and, in turn, causes serious mechanical fracture of the components with deterioration in the device performance. Hence, it is important to introduce self-healability along with good stretchability and conductivity. Inherently self-healable conductive hydrogels and shape adaptive conductive liquid-based designs are proved to be suitable candidates for flexible electronics. A super-stretchable and self-healable TENG electrode with a tensile strain as high as 6000% introduced by Yong *et al.*,^15^ is a good example of a highly stretchable conductive hydrogel. The covalently crosslinked chains of polydopamine (PDA) and polyacrylamide formed a PDA–PAM network rendering super stretchability without mechanical damage. Additionally, healing of different sections of a torn PDA–PMA hydrogel was observed in just 10 minutes without the aid of any external energy or supplementary chemicals.^15^ Polyacrylic acid–gelatin–sodium chloride hydrogel (PAA–Gel–NaCl) electrode, cylindrical rubber–conductive liquid and poly vinyl alcohol–sodium alginate (PVA–SA) ionic hydrogel electrode are all proposed to be quickly healing and scalable systems for self-healable and shape adaptive electrodes.^5,14,27^ The cross-linking network formed by the various components with a continuous conductive trail is the primary reason for their stable performance. Moreover, the patterning of the flexible encapsulation or surface layers of these electrodes contributes to the increase in surface area for better stable electrical output apart from acting as a stretchy protective layer for the liquid electrode. Biomechanical sensors and self-powered wearable devices like smart watches and electronic skin need to be highly transparent and ultrathin along with good flexibility. Transparent elastic substrates embedded with conductive fillers are a good choice for such devices. Here the main challenge is associated with controlling the opacity while incorporating the fillers into the elastic matrix. Uniform conductive layers with thin film-forming capability and lower percolation threshold were also utilized.^12^

## 3. Electrode materials for stretchable TENGs

### 3.1. Stretchable polymeric elastomers and their composites

The prerequisite for a completely stretchable TENG design for wearable electronics is its ability to deal with the complex and arbitrary deformations that occur in frequent human body motions without compromising electrical performance. This calls for the need for extremely flexible and stretchable components that could withstand severe strain cycles and provide long life. Various thermoplastic elastomers and their composites have proved to be suitable materials for fabricating electrodes for fully stretchable and flexible TENGs with excellent power output performance.^16,28^ Generally used polymer matrices include polydimethylsiloxane (PDMS),^29–32^ fluorine groups containing polymers such as polyvinylidenefluoride (PVDF) and Polytetrafluoroethylene (PTFE),^19,33,34^ polyimides,^35,36^ different grades of nylon^37,38^*etc.* Moreover, the ease of processibility, inherent elasticity and soft nature of these polymers help to rely on simple fabrication techniques with good integrity between various layers of the device. Owing to their tunable conductivity and ease of synthesis, conducting polymers such as polyaniline (PANI) and PEDOT:PSS is also utilized for energy storage devices.^13^ Thin films of these polymers in combination with thermoplastic elastomer substrates have played a significant role in developing stretchable TENGs with high conductivity and durability. Several studies have explored the impregnation of conducting polymer films over the pre-strained thermoplastic elastomer substrates for the fabrication of highly stretchable electrodes with good conductivity. Additionally, the feasibility offered by these elastomeric matrices to accommodate different types of 2D and conducting fillers (*e.g.* MXenes, silver nanowires (Ag NWs) and gold nanowires (Au NWs)) to enhance the conductivity of the electrode while retaining other desired properties such as healability, deformability and transparency over a large number of working cycles is also a significant factor in considering them. Also, these fillers could uplift the TENG performance by administering the surface functional groups and work functions.^39^ Consequently, the performance exhibited by these composites in harnessing the mechanical energy from human body motion and converting them to stable electrical signals to achieve self-powered wearable devices has been remarkable. Therefore, proper integration of inherently elastic thermoplastic elastomers with conducting polymers and fillers is of great significance in developing high-performance stretchable electrodes for TENGs in wearable electronics.

**Fig. 5:**
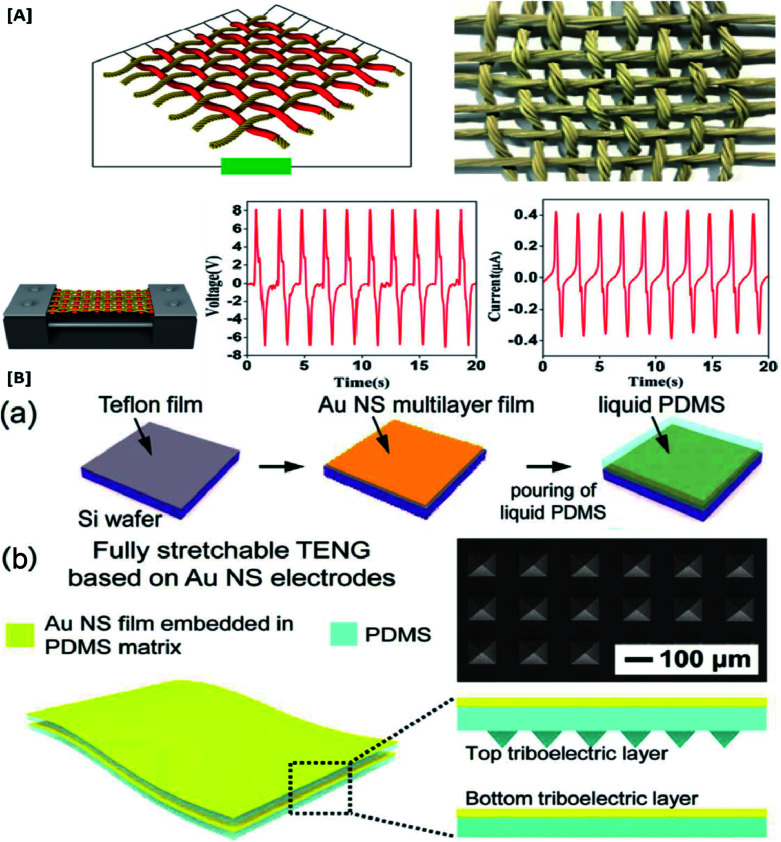
Highly flexible TENG electrodes based on polymer composites: [A] durability and output performance of an all rubber based thread shaped TENG under 100% strain (reprinted with permission,^40^ copyright 2019 Nano Scale Research Letters). [B] Schematic illustration of fabrication of highly durable and fully stretchable gold nanosheets embedded stretchable polymeric matrix based electrode (reprinted with permission,^18^ copyright 2017 Nano Energy).

Significant research has been carried out with different combinations of these polymers and their composites to develop highly stretchable electrodes with maximum power output. Fully rubber-based TENG was developed by Zhu and co-workers^40^ which utilized an ultra-elastic silicone rubber in combination with silver coated glass microspheres to obtain a flexible composite electrode with 100% elongation (Fig. 5A). The electrical output performance and the tensile strength of these stretchable threaded electrodes were evaluated and they were found to have excellent conductivity along with highly stable deformability. An article by Lim *et al.*^18^ reported a highly stretchable TENG device fabricated using normal and micro-patterned PDMS matrices embedded with gold (Au) nanosheets (NSs). In this study, the promising effect of mechanical flexibility of the electrodes in improving the triboelectric output of self-powered TENG devices has been exemplified (Fig. 5B). Moreover, this Au NS–PDMS based electrode tremendously improved the mechanical properties of the device. Excellent output stability was observed for this TENG design (Au NS-TENG) while being subjected to continuous deformation (10 000 cycles) by pushing and stretching. The Au NS-TENGs was successfully applied to the hand joints that can be used in the self-powered human-motion detection processes for wearable applications. This highly stretchable and long-lasting TENG exhibited exceptional electrical stability owing to the intermeshing of Au NS and PDMS which enables to accommodate tensile strain up to 30%. The performance of Au NS-TENGs at repeated cycles of pushing and stretching yielded around 50 V increase in the peak voltage attained (*V*_oc_ increased from 39.4 to 98.9 V) and current from 0.9 to 2.8 μA with contact force ranging from 1 to 6 N. Furthermore, it was noted that the device could be operated using the various mechanical deformations related to the hand movements and thereby a suitable candidate for medical diagnostics and wireless sensors *etc.*^18^

Apart from the complete stretchability, there are certain other criteria like transparency, solvent resistance, self-healability and mechanical robustness expected to be possessed by stretchable TENGs depending upon the applications desired. Most of the polymer matrices are inherently stable at severe conditions and capable of accommodating fillers without compromising on the transparency and stretchability of the composite.^3,8^ A self-charging power system with an inherently stretchable electrode made of carbon black reinforced silicone rubber that can convert deformations into electric charges in conjunction with washable and waterproof silicon rubber encapsulation was developed to directly power electronic watches in a work by Wang *et al.*^17^ Additionally, this power system effectively integrated the TENG component and super capacitor (SC) component to store the harnessed mechanical energy. The hydrophobic nature of the silicon rubber-based outermost surface is responsible for the waterproof nature of the whole design. Transparency is another major requirement of wearable electronics: with functionalities such as touch screens, stretchable materials with excellent transmittance are highly desirable. In a work by Fan *et al.*,^28^ the 78% transmittance achieved by a TENG featuring flexible polyethylene terephthalate (PET) overlaid with indium tin oxide (ITO) over the PDMS layer was observed to be consistently transparent at all regions. Moreover, the exposure of PDMS surface to UV light was found to be favorable for tribocharge generation, which, improved triboelectric charge generation of the TENG.

**Fig. 6:**
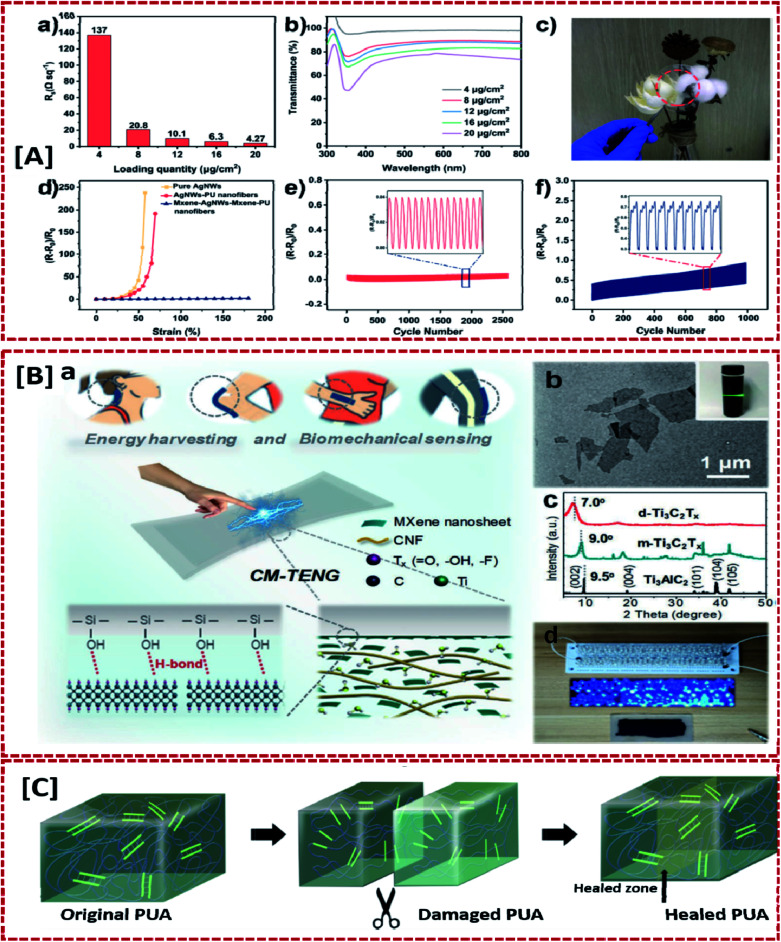
Performance of stretchable TENGs featuring rigid conductive materials in combination with soft polymeric matrices: [A] high performance transparent polyurethane based TENGs incorporated with Ag nanowires and MXene (reprinted with permission,^12^ copyright 2020 Nano Energy). [B] MXene nanosheets based shape adaptive liquid-electrodes for stretchable and wearable TENGs (reprinted with permission,^41^ copyright 2020 Advanced Functional Materials). [C] Schematic illustration of a self-healable and conducting poly urethane acrylate for wearable TENGs (https://doi.org/10.1038/s41467-019-10061-y, open source Nature).

The idea of introducing conducting materials to transparent elastic materials for developing highly stretchable electrodes is also well suited for retaining optical transparency.^12^ In a study conducted by Liu *et al.* initially a highly stretchable PU nanofiber film with a web-like configuration was formed by electrospinning.^12^ The conducting domains of the electrode, MXene nanosheets and silver nanowires were coated over the PU film and then transferred to a PDMS substrate to obtain the hybrid electrode. Here, the MXene component acts as a bridge to firmly attach Ag NWs over the PU nanofibers network, while the transparency and conductivity of the electrode were tuned by varying the concentration of Ag NWs. The PU nanofiber scaffold rendered the stretchability. When tested at a sheet resistance of 10.1 Ω sq^−1^, the fabricated electrodes showed excellent optoelectronic properties with a transmittance of 87.6%. Additionally, even after 1000 deformation-release cycles at a strain rate of 65%, the mechanical durability of the electrode was preserved (Fig. 6A). Furthermore, it was confirmed that this transparent and stretchable TENG device could be used form mechanical energy harvesting and human body motion monitoring applications. This was proved by hand tapping the fabricated device to generate a 38 V peak voltage along with a current density of 1.67 mA m^−2^. The presence of MXene particles favored the adhesion of the conducting material (Ag NWs) to the polymer chains and increased the degree of reinforcement of the particulate fillers in the nanofiber scaffold matrix, rendering excellent output performance and mechanical stability of the device.^12^ One of the major challenges faced by stretchable TENG designs is the delamination of different layers of the device originating from extensive stretching cycles which tend to affect the stability of their electrical output. In work by Parida *et al.*,^16^ a stretchable and healable poly urethane acrylate (PUA) polymer matrix was reinforced with silver flakes as the conductive filler and liquid metal to yield the electrical connection during extreme stretches. This TENG exhibited an unmatched stretchability of 2500% due to the presence of supra molecular hydrogen bonding along with a good conductivity of 6250 S cm^−1^. Moreover, the self-healability and the recovery exhibited after mechanical damage (Fig. 6C) makes this nanogenerator a successful innovation in the area of self-healable energy devices. A highly flexible cellulose nanofiber (CNF) MXene composite based liquid electrode TENG (CM-TENG) was developed by Wen *et al.*^41^ The CM-TENG utilized silicon rubber as the encapsulation layer, exhibited good electrical output performance, which could be attributed to the electron-tapping ability of MXene nanosheets (Fig. 6B). Deformations such as stretching and twisting involved in human body motions could be easily detected by CM-TENGs. On analysing the above-mentioned systems, it could be inferred that the stretchability of the electrodes is attributed to the polymeric component of these systems.^41^ Also, the incorporation of various fillers to these stretchable polymeric matrices generate a conductive pathway and retain the mechanical flexibility of the composite for a large number of strain cycles. Moreover, the materials such as MXene, liquid metals, nanofibers *etc.* added to preserve the integrity between filler particles in the polymer matrix are expected to maintain the electrical connection between conductive materials during various modes of deformation.

Conducting polymer-based stretchable TENGs as devices for harvesting energy for human body motions is also studied extensively in recent research.^13,42^ Various configurations and geometrical designs of stretchable TENG electrodes based on conducting polymers have been explored. Highly stable electrical output and mechanical stability was observed in a PANI coated cotton textile (PANI@WCT) based self-powered wearable TENG. An increase in the time of deposition of the PANI layer was found to enhance the triboelectric output of the system. A maximum open-circuit voltage (*V*_oc_) of approximately 350 V and short circuit current (*I*_sc_) value of approximately 45 μA was obtained under a compression force of 5 N and a frequency of 5 Hz, at a PANI deposition time of 20 h. The mechanical stability of the device was retained even after 2000 cycles of deformation.^13^ Tunable electro-conductivity of conducting polymers is also helpful in modifying the electrical output of TENGs. This property was utilized by Luo *et al.*^43^ in a PANI–PDMS based flexible TENG for creatinine detection. The output performance of this TENG device is depending on the variation in electro conductivity of PANI. The combination of flexibility provided by the Cu/PDMS network electrode film and the electrical response of PANI to the enzymatic reaction of this device is a good example of self-powered sensors in health care systems. A study conducted by Wen and co-workers demonstrated a PEDOT:PSS electrode patterned with a wrinkled surface with considerable stretchability and transparency at a large amount of strain owing to its characteristic constructional design. For the fabrication of WP-TENGs, initially, a PDMS substrate was stretched and then the PEDOT:PSS was coated over it. After drying it was released to obtain a wrinkled PEDOT:PSS electrode over the PDMS which also acted as the triboelectrification/encapsulation layer. Optimum conductivity of 0.14 kΩ m^−1^ and 90% transparency was obtained for the WP-TENG at 100% strain. While the working of the device was assessed on a simulated human skin, the maximum average power was found to be increasing with frequency. It is also explained that the transfer of electrostatic charges from the skin to the PDMS surface occurs during their contact. While the skin moves away and closer to the TENG, the wrinkled electrode aggregates free electrons which eventually flow through the external circuit. Moreover, the output performance of the WP-TENG was improved as the strain level was varied from 0–80%, because of the positive effect of PDMS while stretching. The ability of WP-TENGs to monitor human body movements was also demonstrated in this study. The stretchability and increased contact area of the wrinkled electrode at different bending angles of the human body motion led to increased output voltage with good repeatability over several cycles. Apart from that, the WP-TENG also showed high performance active tactile sensing properties which occurs because of increased contact area between elastomeric and dielectric materials under pressure. Frontline applications such as wearable electronics and e-skins could undoubtedly rely upon this promising design for better performance.^3^ In another work by Shi *et al.*,^42^ a stretchable, liquid electrode TENG was developed, in which liquid form PEDOT:PSS was as the electrode and silicone rubber as the triboelectrification layer as well the device encapsulating material. To fabricate the TENG, a grooved acrylic model with a cavity engraved by laser cutting for injecting PEDOT:PSS was made. After that, the silicone rubber was poured into the acrylic channel and eventually a hollow template of silicone rubber with Al foil as the lead wire was formed. Following this, the liquid PEDOT:PSS was introduced into the cavity to obtain the desired PL-TENG. The generated *V*_oc_, *I*_sc_, *Q*_sc_ and average power of 265 V, 24.9 μA, 85 nC and 24.8 μW at a frequency of 2.5 Hz under single electrode working mode is a clear manifestation of the suitability of the designed PL-TENG to be used to sufficiently power wearable electronics from human body gestures. This flexible PL-TENG with good conductivity also exhibited a sustained electrical output performance even after being subjected to severe conditions of washing, prolonged storage and various modes of deformation.

### 3.2. Inorganic and ceramic materials and their polymer composites

**Fig. 7:**
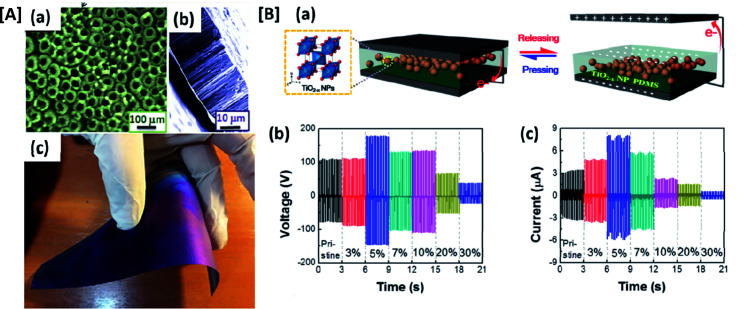
Inorganic semi conducting material based stretchable electrodes for wearable TENGs: [A] photograph and microscopic image of a self-assembled TiO_2_ nano arrays based flexible conducting active electrode for TENGs (reprinted with permission,^44^ copyright 2017 Advance Engineering Materials). [B] Voltage and current output performance of a TiO_2_ nanoparticles embedded PDMS matrix (reprinted with permission,^32^ copyright 2018 Micromachines).

The recent focus in the field of materials for flexible TENG electrodes is on inorganic metal oxides, ceramic materials and their polymer composites. The suitability of inorganic materials as stretchable electrodes for wearable electronics, besides the extensively utilized inherently flexible polymeric materials, is exemplified in recent studies. Most of the inorganic semiconductors, metal oxides, ceramic materials and their composites have shown comparable performance to that of organic fillers and their elastomeric composites. Self-organized TiO_2_ nanorod arrays over flexible titanium substrate as active electrode material (Fig. 7A) were demonstrated by Raheleh and co-workers^44^ for TENG and its electrical output has been compared with that of a flat TiO_2_ electrode. The nanostructured arrays of TiO_2_ improved the efficiency of TENG, yielding a high output voltage of 40 V and a current density of 1 μA cm^−2^. The enhanced performance of a nanotubular array of TiO_2_ (*i.e.* compared to flat TiO_2_) is due to the increased charge accumulation observed in their nanostructured interface and increased internal contact area. The presence of nano arrays on both sides of the electrodes favoured an increase in roughness of the surface as compared to flat TiO_2_ electrodes and thereby, yielded higher values of output current and voltage resulting from higher charge transfer.^44^ The capability of TiO_2_ based stretchable nanogenerators in charging a 683Nf capacitor within 6 to 12 s to a maximum output voltage of 75 V, also shows the suitability of these electrodes to be used as sensors and self-powered systems. Apart from that, the hydrophilicity of TiO_2_ and the changes observed in its charge transfer due to adsorption of water molecules could be utilized to design TiO_2_ based TENG electrodes for humidity sensing.^45^ In addition to the self-assembled systems, embedded structures in suitable polymer matrices are also developed for stretchable electrodes. A TiO_2_ embedded PDMS composite based stretchable electrode was designed by Park *et al.*, for TENGs (Fig. 7B).^32^ These composites utilize the excellent dielectric properties of TiO_2_ and the inherent flexibility of the PDMS matrix to show better output voltage and current. The change in oxygen vacancies of the PDMS surface and the enhancement in dielectric constant was observed to be maximum at a TiO_2_ loading of 5%, which is responsible for the excellent output performance. However, above 5%, uniform dispersion of TiO_2_ in PDMS matrix was disturbed forming nanoparticle aggregation; thereby, degrading the performance of the TENG. Generally, the electrochemical anodization technique employed for the fabrication of self-assembled TiO_2_ nano arrays over titanium substrates provides good stretchability to the electrodes. The feasibility of engaging such fabrication techniques in the case of inorganic materials is an advantage of such materials over flexible polymeric substrates. Additionally, the nanostructured surface modification made possible by the anodization technique also favors good surface charge density without further treatment of the electrode surface. Moreover, in the case of self-assembled nano-structures, unlike in elastomeric substrates, the requirement of incorporation of conductive fillers could be ruled out. This, in-turn, would help to avoid the issue associated with percolation networks and opacity. When it comes to embedded metal oxide nanoparticles in polymeric matrices, the optimization of the weight ratio of the fillers in polymer matrix is crucial. This is because, above the optimized loading percentage, the aggregation of filler occurs leading to the formation of a separate electron path of leakage current. Also, the formation of oxygen vacancies observed specifically in metal oxide nanoparticles provides electrically positive charges. This enhances the exchange and trapping of electrons under the working condition of the TENG assembly.

Ferroelectric polymer matrices incorporated with inorganic particles shows enhancement in dielectric polarization and thereby increasing the triboelectric surface charge density. In a study conducted by Kim *et al.*, two different ferroelectric polymers were incorporated with varying concentrations of MoS_2_ flakes for triboelectric surfaces of opposite polarity. The opposite tribo polarity of MoS_2_ nano flakes composited nylon 11 and PVDF matrices which were used as the frictional layers improved the surface charge density. Additionally, the dipolar polarization exhibited in the triboelectrification layers owing to the ferroelectric properties of the selected polymers, further enhanced the performance. Moreover, this TENG has exhibited an 8-fold increase in output current and voltage as compared to that with nylon 11–PVDF layers without MoS_2_ flakes.^46^

### 3.3. Carbonaceous materials, other metallic nanostructures, and their composites

Graphene as electrode material in TENGs has recently garnered interest owing to its tunable and high electrical conductivity, optical transparency,^47^ gas barrier property, robustness flexibility, favourable work function,^48^ and environmental stability and other mechanical properties.^49–51^ Tian and co-workers^52^ fabricated graphene oxide thin film electrodes to be used in nanogenerators due to their peculiar electrostatic properties. Since then, graphene along with its oxide (GO) and reduced oxide (RGO) forms had been extensively investigated for use as electrodes in both piezoelectric^53,54^ and triboelectric nanogenerators. The first graphene-based electrode for TENGs has been developed by Kim *et al.* in 2014.^55^ Even though the TENG devices utilizing these CVD-grown graphene (grown on Cu or Ni foils) electrodes exhibited less output current density, it is considered as a promising material for TENGs due to the facile fabrication processes, cost-effectiveness and flexibility. Additionally, the studies on graphene-based electrodes for TENGs also emphasize the optimization of the properties of graphene through surface functionalization.

**Fig. 8:**
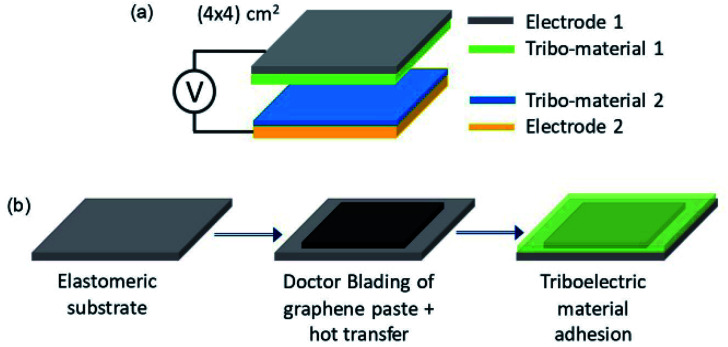
(a and b) Schematic illustration of the fabrication of a graphene based stretchable electrode by embedding the graphene layer over an inherently stretchable elastomeric substrate operated in a vertical contact separation mode (reprinted with permission^56^ copyright 2020 Nano Energy).

Few-Layer Graphene (FLG) based electrodes (Fig. 8) for TENGs were developed from FLG flakes through a wet jet milling technique in a study conducted to develop graphene-based flexible electrodes.^56^ The obtained paste of graphene was coated on ethyl vinyl acetate as a film for doctor blading and was combined with porous polyamide 6,6 (nylon) and PVDF membranes. The advantage of using such wet and ambient processes is the high flexibility of the obtained electrode and the feasibility of process scale-up. The second tribo-electrode used is e-beam evaporated gold electrodes. The work also included the comparison of two different types of arrangements in vertical contact separation mode TENG operation *viz.*: Au/nylon//PVDF/Au, FLG/nylon//PVDF/FLG. The electrical conductivity of electrodes was measured using the van der Pauw method^57^ and found that it is four times higher for FLG than Au electrode. However, the difference in power density generated was found to be 26 times higher than for Au electrodes. The increase in output power density of the FLG incorporated TENG was attributed to its higher surface roughness and higher electrode capacitance. The study was also extended to a comparison of power density obtained when functionalized Au (Au–NH_2_ and Au–CH_3_) electrodes are used instead of Au electrodes. The higher power density exhibited by FLG electrodes was attributed to lower work function when compared to Au and Au functionalized electrodes.

A similar surface modification strategy was reported by Chen, *et al.*,^58^ in synthesizing crumpled graphene (CG) surface, which was utilized to develop a TENG with high power density (up to 20 fold greater than planar graphene-based TENG). Additionally, a higher output current of 25.78 μA was achieved by connecting PMMA/VHB/CG and PDMS, with an approximate triboelectric area of 2 × 2 cm^2^ and a space gap of 5 mm. These advantages were due to the presence of a rougher surface and greater surface area. The work function of graphene was regulated during crumpling, and the stretchability was increased tremendously such that the TENG was able to withstand a strain of 120% without deformation due to continuous working and a power density of 0.25 mW cm^−2^ was observed. The extension of the study was reported by the same group in 2020 where the crumpled graphene was further doped with AuCl_3_.^59^ It was observed that when 200% crumpled graphene was doped with AuCl_3_ (spin-coated on the CG) of concentration 1.2 mg mL^−1^ of water, the output voltage and current were raised to 80.6 V and 11.9 μA cm^−2^.

Despite its excellent conductivity and transparency, the use of graphene was limited due to the high tensile strength which resulted in a fracturing of the electrode at low strain (less than 5% strain). Hence the surface modifications and functionalization of graphene is an area of great interest to enable the extensive use of graphene for nascent stretchable and wearable electronics applications.^60^ Yang, *et al.*^61^ used graphene composites as electrodes in TENGs to harvest the ubiquitous kinetic energy and utilize it as self-powered sensors to monitor human joint movements such as finger gesture sensors. The study emphasizes enhancing the electrical conductivity and surface wettability of graphene by compositing it with the conductive polymers poly(3,4-methylenedioxy-thiophene):polystyrene sulfonate (PEDOT:PSS). These electrodes not only yielded better output current density and power (enhanced by 140%, output current density of 2.4 μA cm^−2^) but also exhibited high electrical and mechanical durability. The PEDOT:PSS was spin-coated on the CVD grown graphene film which was transferred onto PET. The studies on surface modification and the electrical properties analysis of the electrodes affirmed the enhanced performance of the electrodes.

The conformity of TENG surfaces with human skin is important when it comes to wearable electronic devices like real-time BP monitors and other skin attachable devices.^62^ The fabrication of conformal graphene electrodes in a single electrode TENG using PET (polyethylene terephthalate) as the substrate layer and PDMS as the electrification layer was explained by Chu *et al.*^63^ This TENG exhibited better conformity, as all the materials used for fabrication were thin and with low bending stiffness. Consequently, the unit which has a thickness of ∼2.4 μm rendered good flexibility as well as compatibility with the rough human skin. Moreover, the PDMS encapsulation layer was subjected to plasma treatment for enhancing the surface area without compromising the output power density. A considerable increase in *V*_oc_ and *I*_sc_ was obtained through the formation of surface microstructures as a result of the plasma treatment.

The use of laser-ablated graphene (LAG) in TENG based pressure sensors (TEPS) with high sensitivity was reported in 2019.^64^ The LAG was fabricated in a single step *via* a laser scribing technique on a thin and extremely sensitive Kapton (PI 55 μm) film. The proposed TENG was operated in contact separation mode. A combination of micro structured PDMS (PDMS mixed with cross-linker poured onto sandpaper and spin-coated to obtain 200 μm thick PDMS film) on LAG acted as one tribo-layer a PET/ITO (polyethylene terephthalate/indium tin oxide) layer acted as the working structure for TENG. Also, these triboelectric layers were sandwiched between two acrylic layers to increase the mechanical strength of the unit. This investigation reports an exceptionally low response time of 9.9 ms. The TEPS gave a stable response on repeated trials which make it useable as a self-powered human gesture detector as well as a finger pulse monitor. These studies in which graphene was modified through different strategies to be incorporated as a TENG electrode, gave a new vista for researchers and led to further development of electrode materials.

Graphene electrode (Laser-Induced Graphene (LIG)) was fabricated similarly in one of the studies on flexible electrodes by Stanford, *et al.*^35^ It was observed that the non-carbonaceous elements were degassed when exposed to laser and high temperature of 2500 °C and also the synthesized graphene obtained exhibited improved surface area and porosity. The major advantage of this method was the feasibility of using a commercially available laser cutter for electrode fabrication and the possibility of using any carbon source including chemicals like PI and natural materials like wool and cotton, *etc.* The materials characterizations confirmed the flaky structure of the film which makes it flexible and appropriate for use in wearable electronics. The fabricated electrode film was used in a single electrode triboelectric nanogenerator (SETENG) consisting of a negative LIG/PI friction layer and aluminium as the positive triboelectric layer. The obtained *V*_oc_ and *I*_sc_ of the TENG were 35 kV and ∼60 μA respectively from a contact surface area of 36 cm^2^, which is very well compared with the results observed in the literature. Thus, this simple inexpensive fabrication method is promising for the production of TENG based wearable electronic systems. LIG was deposited on both tribo-negative and tribo-positive materials in a study reported by P. Zhao and co-workers.^65^ The additional adhesive interface which is usually present while metal electrodes are used can be avoided in this case (*i.e.*, contact impedance is lower in this case). An increase in output (maximum output power 2.25 W m^−2^, current density of 20 mA m^−2^) is attributed to the decrease in contact height. It can be inferred that not only the electrode material but also its surface and the way of contacting with the triboelectric layers are also important parameters to be considered while fabricating a TENG.

Shear exfoliation of graphene with the aid of water is the electrode fabrication method employed in a study reported by Shin *et al.* in 2018.^66^ The major advantage of this method is that the process is easily scalable and cost-effective as a non-toxic, inexpensive solvent is used unlike in the cases where reduction of graphene oxide is required to make it conduct. Moreover, the graphene thus produced is easily transferable to any substrate *viz.*; glass, silicon wafer, paper, *etc.*, therefore, it is an excellent method for the fabrication of electrodes for wearable electronic applications like e-skin.

The use of graphene oxide (GO) for electrodes has gained prominence due to the oxygen functional group and higher surface area (compared to graphene). It is observed by Ejehi *et al.*^67^ that GO in TENGs give better results when employed as self-powered humidity sensors because of the interaction between free oxygen functional groups and the water of water. The GO paper (32 μm thickness and surface area of 50 cm^2^) was fabricated by both the Hummers' method and drop-casting to function as an electrode. In both types of TENG, the impedance or capacitance was noted to measure the relative humidity (RH), which is expressed as a function of output voltage. As the water molecules get adsorbed to the GO paper surface, there is a difference in output voltage observed, whereas there was no variation in current observed. The characterization studies confirmed the absence of stacking of GO sheets, hence this TENG is acclaimed as the first nanogenerator using GO paper which displayed a higher power density of approximately 1.3 W m^−2^ at 2 Hz frequency. It was also noted that, at 2 Hz frequency, when the RH was raised the open-circuit voltage reduced gradually from 144 V to 14 V.

Carbon nanotubes (CNT) have also generated interest as electrode materials for TENGs due to their exceptionally high tensile strength, optical transparency, chemical stability, and low weight due to their structure.^68,69^ These properties of CNTs are attributed to the presence of sp^2^ bonds^70^ like in graphene. The incorporation of CNTs in TENGs for the fabrication of wearable electronics is reported in various literature. In the work reported by Ohno *et al.*^71^ the CNT is working with the dielectric PDMS layer which is further surface modified with CF_4_ plasma. The maximum output power density is reported to be 14.3 W m^−2^. The increase in output power density is attributed to two reasons *viz.*, the increase in electronegativity of PDMS due to the presence of the fluoride functional group and the characteristics of CNT such as excellent crack resistance against repeated cycles of strain. The optical transmittance of the material is 95% which makes it an appropriate choice for wearable electronics.

The fabrication of vertically aligned carbon nanotubes (VACNT) as an electrode for TENG was reported by Oguntoye and co-workers.^72^ The higher stability exhibited on repeated cycles of compression and better mechanical properties makes this vertically aligned arrangement preferable. The TEM image of the VACNT showed that they are made up of multi-walled carbon nanotubes (MWCNT) and are grown up to a length of 1 mm. It is observed that the maximum *I*_sc_ and *V*_oc_ obtained in a VACNT–PTFE system is 0.21 μA and 3.2 V respectively and in VACNT–PET system is 0.16 μA and 1.42 V respectively from a contact area of 1 cm^2^. Similarly, Hong *et al.*^73^ reported a study where laterally combed CNTs were fabricated to overcome the intrinsic properties of CNTs such as deterioration while stretched, lower conductivity compared to metals, *etc.* The TENG unit fabricated in this study displayed an optimum power density of 33.5 mW m^−2^. Therefore, it can be ascertained that CNTs, when modified appropriately, can be a prospective material in the field of stretchable electronics and energy storage.

The use of MWCNT as a coating on cotton knitted fabrics to function as the electrode of a wearable TENG is stated in work by Shi *et al.*^74^ which is focused on developing a “power shirt”. The TENG thus developed is working in single-electrode mode. The functioning unit consists of three layers *viz.*; dielectric 1, dielectric 2 and the conductive fabric layer. Due to the strong chemical interaction between the cotton fabric and MWCNTs, the conductivity and the flexibility of the electrode is higher than in other wearable TENGs. The method used for fabricating the electrodes is dip coating of commercially available cotton fibre in a dispersion of MWCNT in deionized water containing sodium dodecylbenzene sulfonate followed by drying. This procedure was repeated to get an even denser coating on the fibre. It was noted that the peak output power density of ∼12 μW cm^−2^ was obtained from a unit of 9 cm^2^ area. It was found that the resistance of the electrode decreased considerably with increasing iterations of dip coating which can be due to the increase in adhesive force between the fabric and MWCNT and the subsequent continuous path for the flow of charge.

Enhancement of output power density using composite electrode fabricated by incorporating nanoribbons of reduced graphene oxide nanoribbons (RGONRs) in polyvinylidene fluoride (PVDF) matrix was reported in 2016.^75^ The improvement in voltage and current of the TENG is attributed to the combined effect of properties of PVDF and RGONRs, like high aspect ratio, and oxygen-carrying functional groups which increase the electronegativity of the electrode.^76^ It can be found that the oxidation of RGONR due to the compositing has increased the surface roughness compared to that of pristine RGONR. The positive electrode in the arch-shaped TENG was an aluminium film (which also functions as the triboelectric surface) and was grounded. The peak output obtained is 0.35 V using this TENG from the input of mechanical movements of frequency below 10 Hz.

Li *et al.*^76^ in their work fabricated a vertical contact separation mode TENG where Cu NWs/RGO (copper nanowires/reduced graphene oxide) electrode was used instead of conventional electrodes. The electrode was fabricated using the one-pot method where cuprous chloride, ammonium chloride and oleyl amine was added to the homogenized solution of graphene oxide dispersed in ethyl glycol, glycerol and polyethylene glycol and allowed to react for twelve hours in a hydrothermal synthesis reactor at 200 °C. The synthesized CU NWs/RGO was further processed to make electrode films for TENGs. It was noticed that the lifetime and conductivity of Cu NWs/RGO electrodes were higher than those of pristine Cu NWs electrodes as the graphene layer prevents the oxidation of Cu nanoparticles. This composite electrode also displayed excellent optical transmittance on both PET and PDMS substrate in the wavelength range of visible light. A comparison of optical transmittance of Cu NWs/RGO with the commercially available PET–ITO film electrodes is also discussed and it is seen that the transmittance of Cu NWs/RGO electrodes was 70% higher than that of commercial PET–ITO electrodes. Therefore, the transparency of the TENG is not compromised due to the modification of the electrode. The properties such as flexibility and stability on repetitive use of the unit are also mentioned to be good which makes it appropriate to be used in electronic skin and other flexible and transparent devices.

Similar hybridization is employed for graphene in a work reported by Vaka and co-workers.^77^ The electrode was fabricated with functionalized AuNPs sandwiched between graphene layers. The layered structure of the electrode makes the transport of charge easier due to the formation of conducting pathways. The study also indicated a higher sensitivity (5 × 10^−4^ kPa^−1^, response time < 15 ms) of the sensor (within a pressure range of 86 × 10^−3^ to 539 × 10^−1^ kPa), thus fabricated which makes it ideal for application in e-skin.

Even though electrodes based on carbon-based nanomaterials are cost-effective, the poor optoelectronic performance creates dissatisfaction compared to conventional transparent and flexible electrode material like indium tin oxide (ITO). However, alternatives for ITO-based electrodes are needed owing to the surplus material waste generation, expensive fabrication procedure, inclination to cracking due to brittleness, and therefore it is unable to withstand bending or rolling. Furthermore, ITO being a rare material is highly expensive as well.^78^ Conversely, noble metals such as Ag nanowires and gold (Au) films which possess low sheet resistance (*R*_s_) are capable of surpassing ITO and hence are well-recognized electrode materials.^79^

The high surface area possessed by metallic nanostructures boosts the electrical conductivity of the TENG electrodes.^80^ Deposition of these materials on transparent and stretchable polymers (PDMS, silicon rubber, *etc.*) highly favor their use as reliable flexible electrodes in TENGs. In a study to develop touch screen products in an affordable way, where energy is harvested *via* touching, pushing, striking, *etc.*, researchers proposed to fabricate TENG electrodes devoid of the typical coating of metal electrodes on triboelectric material films. They designed a flexible single-electrode triboelectric nanogenerator by depositing a semi-cured PDMS thin layer on Ag NWs as the versatile electrode where the influence of a friction effect between PDMS and Ag NWs improves the TENG performance.^81^ Another promising category of transparent conductive electrodes is copper nanowires because of the good optical transparency, better electrical conductivity, rich reserves, and affordability shown by them. Nonetheless, the high oxidation rate of Cu NWs hinders their wide applications. Lately, a group of researchers solved this problem by encapsulating Cu NWs with rGO. This group developed a UV curable composite resin of single-crystal graphene and Cu NW/which showed high optical and electronic output stability with a significant oxidation resistance (Δ*R*/*R*_0_ < 0.2 within 180 days) compared to pristine Cu NWs (Δ*R*/*R*_0_ > 1 after 1 day) or polycrystalline graphene-covered Cu NWs (Δ*R*/*R*_0_ > 1 after 7 days) (Fig. 10).^82^ Similarly, Li *et al.*^76^ developed a transparent triboelectric nanogenerator with high flexibility and output by crafting the Cu NWs/RGO composite electrodes *via* one-pot synthesis to prevent the oxidation of Cu NWs and the uniform distribution of the graphene layers covering Cu NWs amplifies the conductivity of the nanowires.

**Fig. 9:**
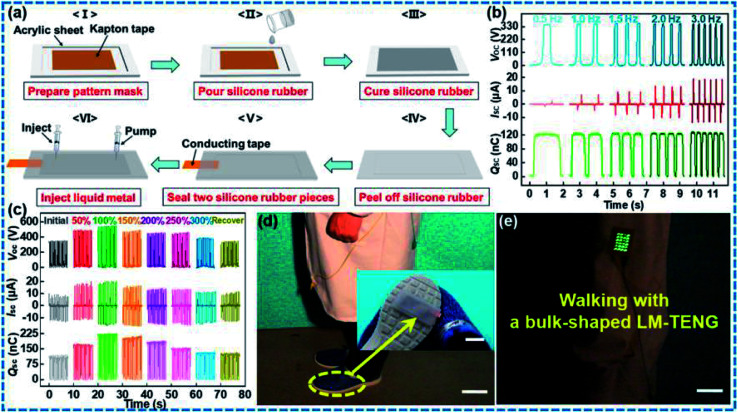
(a–e) Schematic illustration of the fabrication of a fully transparent and stretchable PDMS based liquid metal electrode and its application as a motion tracker in human body movements (reprinted with permission,^106^ copyright 2018 ACS Nano).

**Fig. 10:**
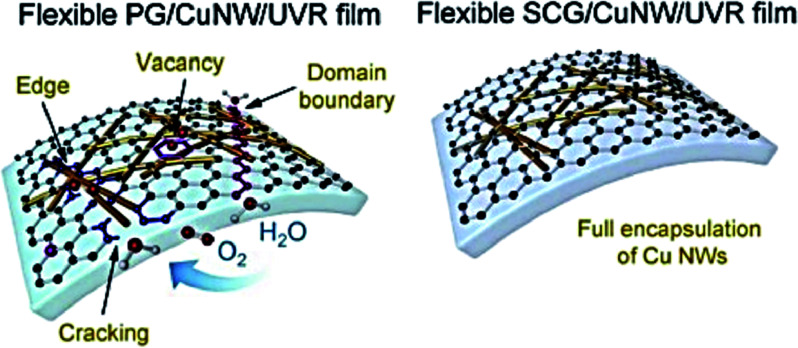
Schematic diagram of the flexible SCG/Cu NW/UVR and PG/Cu NW/UVR films illustrating the network of copper nanowire (reprinted with permission,^82^ copyright 2020 Nano Energy).

The scope of energy harvesting from human movements *via* wearable TENG is widely accomplished by integrating nanogenerators into clothing or accessories and is developed into textile-based TENG encompassing distinct motifs and applications is mainly comprised of fibre, yarn, or fabric-based TENG patterns. Innovative and flexible electrodes are inevitable to counter practical challenges which question their durability other than efficiency. Hence, significant attention has been directed towards developing textile-based TENGs. In a recent work by Dong *et al.*,^83^ silver-plated nylon yarn electrode-based shape adaptable and resilient braided TENGs as e-textiles have been developed. The resulting e-textiles exhibited good structural integrity, cyclic washability, and mechanical stability. When the cost-effective silver-coated bulk nylon braided yarns are used as the outer electrode in the multiaxial winding yarn instead of extremely thin and expensive silver nylon single thread, they were found to be capable of harvesting vibrational energy and sensitive to the slightest weight alterations. Hence, this is a promising candidate for applications like pressure sensing, human motion monitoring intelligent shoes, and self-powered identity recognition carpet for security purposes.^83^ Zhao and co-workers^84^ coated Cu on polyethylene terephthalate yarns and fabricated Cu-coated PET (Cu–PET) warp yarns *via* direct weaving which acts as both electrode and tribo-contact material. Copper holds superior strength and wear resistance over silver, is easily available and affordable, and is a typical base metal for electrode applications.

The innate features of metals including high electrical conductivity, stability, and ease of optimized device engineering favour the development of efficient electrodes in the arena of energy harvesting. However, the weakness from the thickness and the corresponding high-density is tackled by introducing many structural architectural designs and optimizations such as using physically or chemically deposited thin metal films on flexible substrates or embedding metal nanoparticles in elastomeric substrates, *etc.* Furthermore, such flexible ultrathin metal electrodes prove to have high strain endurance and stress absorption without deformation during rough stretching and bending conditions, thus finding practical applications in stretchable and wearable electronics.

It is understood that the major requirement of the electrode materials for TENGs for wearable application is their stretchability, which demands the use of inherently stretchable materials or a combination of rigid and soft materials. Mainly thermoplastic polymers such as PDMS, nylon, PUA *etc.* and their composites with suitable fillers are effective in fabricating highly stretchable electrodes (Table 1). Additionally, thin film-forming polymers such as PEDOT:PSS and PANI with inherent conductivity coupled with flexible substrates are also found to be a reliable design for fully stretchable electrodes. Moreover, these structures retain important properties such as transparency which is crucial in the case of wearable applications. The electrode properties could be further enhanced if conductive fillers such as metallic nanoparticles are added to the conducting polymers. Here the output properties of the electrodes could be further enhanced, retaining the mechanical robustness of the whole device. Liquid metal and polymeric hydrogels are found to be promising materials for developing deformable and mechanically stable electrodes for stretchable TENGs. The cross-linked structure of these hydrogels and their shape adaptive nature provides self-healable designs which could retain the stretchability of the electrodes for a large number of working cycles. Also, the rubbery outer layer used in such designs is suitable for protecting the hydrogels from harsh surfaces and extreme deformations. The inorganic semiconductors and ceramic materials organized over suitable patterned substrates have resulted in stretchable active electrodes with excellent output performance. The basic nanostructured assembly of these materials favours better charge transfer and contact area in addition to good flexibility. However, in the case of composites of these inorganic filler materials, agglomeration of the reinforcing particles is to be considered, which would otherwise lead to deterioration of the output current through leakage of electrons. On the other hand, graphene-based stretchable electrodes have also been extensively used in wearable devices. The combination of graphene electrodes with conducting and thin film-forming polymers such as PEDOT:PSS was utilized to develop highly conducting stretchable electrodes with excellent current density and power output. Moreover, various surface modifications given to these electrodes helped to enhance the surface roughness and maintain the continuity of conductive pathways. The higher stability and light weight of highly conductive carbonaceous materials such as CNTs when embedded in stretchy polymeric matrices such as PDMS were found to yield excellent output power density. Apart from that, attributes such as excellent crack resistance, tunability of their alignment *etc.* help to improve the number of working cycles of the TENG electrodes, without compromising on flexibility. Finally, the right choice of the soft and flexible polymeric film along with optimized levels of conductive fillers seem to be the most promising combination for stretchable electrodes for wearable TENG applications.

**Table 1:** Summary of different electrode materials and their output performance used for wearable TENG devices

TENG system	Material(s) used	Output performance	Application(s)	References
Stretchable all rubber-based thread shaped TENG	Silver coated glass microsphere/silicone rubber	*V*_oc_ = 3.82 V	Biomechanical energy harvesting (human joint motions)	40
		*I*_sc_ = 65.8 nA (at 100% strain)		
Fully stretchable and durable TENG	Au nanosheets embedded PDMS matrix	*V*_oc_ = 98.9 V	Medical diagnostics and wireless sensors	18
		*I*_sc_ = 2.8 μA (at 6 N force)		
Self-charging, waterproof SC-TENG	CB electrode/PVA based material	*V*_oc_ = approx. 400 V	Electronic watch	17
		*σ*_sc_ = approx. 97 μC m^−2^		
Stretchable, transparent TENG	MXene nanosheets, silver nanowires, PU, PDMS	*V*_oc_ = 38 V	Motion sensor	12
		Current density = 1.67 mA m^−2^		
		Max. strain = 180%		
Stretchable, healable TENG	Ag flakes/PUA	Stretchability = 2500%	Deformable energy harvesters and 3D printing	16
		Conductivity = 6250 S cm^−1^		
Highly flexible TENGs	TiO_2_ nanorod arrays, titanium substrate	*V*_oc_ = 40 V	Biosensors, photo detectors	44
		Current density = 1 mA cm^−2^		
Enhanced stretchable 2D material based TENG	Crumpled graphene	*V*_oc_ = 83 V	Wearable applications	58
		*I*_sc_ = 25.78 μA		
		Power density = 0.25 mW cm^−2^		
Coated fabric TENG	Polyaniline coated cotton	*V*_oc_ = 350 V	Wearable textile TENGs	13
		*I*_sc_ = 45 μA		

## 4. Fabrication strategies for stretchable TENG electrodes

### 4.1. Geometrical patterning of conductive materials

**Fig. 11:**
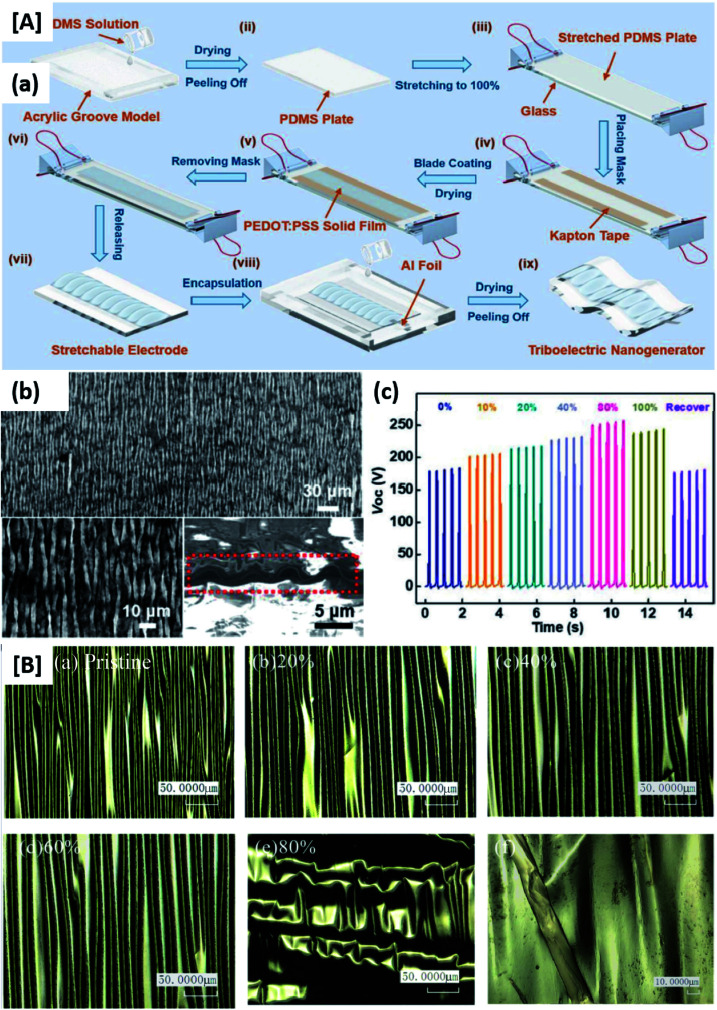
[A] (a) Schematic diagram of a fabrication process flow of the stretchable and transparent wrinkled PEDOT:PSS film-based triboelectric nanogenerator (WP-TENG). (b) SEM image of a wrinkled PEDOT:PSS film on an elastic PDMS substrate. The left bottom inset shows an enlarged wrinkled morphology. The right bottom inset shows the cross-section view of the wrinkled film. (c) *V*_oc_ of the WP-TENG under various tensile strains ranging from 0 to 100% (reprinted with permission,^3^ copyright 2018 Advanced Functional Materials). [B] Morphologies of the PEDOT:PSS with 100% wrinkled degree during the tensile process: (a) pristine condition, (b) 20% strain, (c) 40% strain, (d) 60% strain, and (e) 80% strain (scale bar: 50 μm). (f) The micrograph of the micro-crack (scale bar: 10 μm) (reprinted with permission,^87^ copyright 2018 Advanced Functional Materials).

Several investigations are being undertaken in the recent studies focused on the development of device fabrication and designing strategies for TENG components that can fulfil the goal of industry-standard flexible TENGs. Various geometrical designing and patterning strategies have been investigated recently for rendering stretchability to rigid conductive materials that can be used in wearable TENGs. This can increase the rate of strain deformations and mechanical durability of these materials without compromising their conductivity. Especially, rigid conductive materials which are carbonaceous, metals and metal oxide nanoparticles and thin films of conducting polymers are investigated for developing highly stretchable and conducting electrodes for stretchable TENGs. In this section, various possible surface geometrical design strategies are introduced. Yang *et al.*^85^ developed a TENG with serpentine patterned Cu electrodes for accompanying stretching and compression. The fabrication procedure involves two-step sputtering, in which the first step utilizes a serpentine mask and a second step without the mask, on a PDMS substrate. The serpentine electrode maintained a stable and lower resistance over 10% of tensile strain instead linear electrode that underwent break at 6% tensile strain. Unlike their wide usage of inflexible electronics, TENGs are underutilized in applications where optical transparency is required. Though there are tribomaterials with transparent properties, electrodes with considerable transparency, conductivity and easily patternable are least reported. Song *et al.*,^86^ successfully deposited patterned Ag NWs using electrospray deposition with grounded electrolyte solution (EDGE). The technique allows the selective deposition of Ag NWs on grounded electrolyte solution of specified pattern, reducing overall Ag NW leakage elsewhere. The low wastage of Ag NWs, variety of substrate selection, 3D surface compatibility and optical transparency *etc.* are the advantages of this fabrication technique. Single electrode mode is enabled in patterned electrodes and tactile sensors are demonstrated using PDMS and PMMA substrates as tribo materials. Among the transparent electrodes PEDOT:PSS is extensively studied owing to its flexibility and conductivity. However, structural modifications are less explored. Chen *et al.*,^3^ developed a buckled PEDOT:PSS structure for withstanding continuous stretching. The buckled structure is produced by spin coating of the PEDOT:PSS on the stretched PDMS film. On releasing the system PEDOT:PSS achieves buckled or wavy structure which ensures high order of stretchability. The PEDOT:PSS film has also performed high conductivity and wettability by the incorporation of Zonyl doped solutions. The same fabrication method was adopted by Y. Xiao *et al.*^87^ for the demonstration of a wearable electronic device by the deposition of PEDOT:PSS film on stretched PDMS film by blade coating technique. Single electrode mode was employed for the energy harvesting from the biomechanical motion and tactile sensing. As explained above, the PEDOT:PSS system has been well studied as a flexible transparent electrode as well its ability to form buckled structures with simple procedures. However, the system is not employed for the simultaneous detection of strain and pressure. Xiao *et al.*,^87^ developed micro-crack and wrinkled PEDOT:PSS based TENG for pressure sensing. The resistive behaviour of the device is demonstrated for strain sensing. The wrinkled patterns are effectively incorporated on the conducting polymer by a roll coating rod on a stretched high bond tape followed by external strain and shrinkage. The external strain initiates micro-cracks in film during the fabrication procedure causing resistance change while measuring strain (Fig. 11B). Also, wrinkled structure enhances the effective surface area and enhanced triboelectric output. Similar wrinkled micro/nano structures are extensively employed in tribomaterials as well as electrode materials since the structure can work as tribomaterials as well as a spacer.^88^

**Fig. 12:**
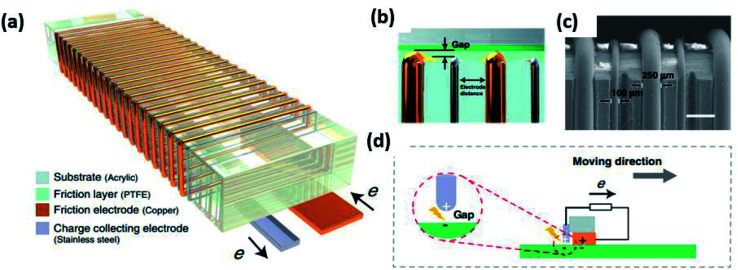
Design and working mode of MDC-TENG: (a and b) schematic and (c) SEM image of MDC-TENG (scale bar: 500 μm). (d) Schematic diagram of DC-TENG working mechanism (https://doi.org/10.1038/s41467-020-20045-y,^89^ open source nature).

Significant research has been carried out in the microstructure patterning of electrodes for high-performance triboelectric performance. Normal modes of a TENG give rise to AC which required rectification before energy storage or smart device powering. However, in a TENG electrostatic breakdown between triboelectric materials leads to the formation of DC. Zhao *et al.*^89^ developed a DC TENG based on rationally patterned Cu and stainless steel (SS) microwires to get enhanced output (Fig. 12). The wires are consecutively placed around an acrylic substrate and a PTFE is allowed to slide over the electrodes maintaining a small gap at the SS electrode to produce electrostatic breakdown. This structural novelty with 50 micro-patterned wires in a 1 cm scale exhibited a 10-fold increment in the output performance compared to previously reported DC TENGs. The miniaturization of electrode structure resulted in the surface charge density enforcement and strong breakdown resulted in 4.4 mC m^−2^.

The fabrication of electrodes is a significant part of a triboelectric nanogenerator as proper electrodes support the charge transfer, smoothness, flexibility of TENG *etc.* In that regime different type of patterning has attained considerable attention nowadays. The patterning on flexible electrodes with scalable and industrial standard technologies is the best way to choose for a TENG fabrication. Chen *et al.*^90^ demonstrated laser-induced graphene (LIG) 3D patterning on PI substrate with Au nanoparticle embodiment for hybrid material development. The fabrication procedures include CO_2_ laser irradiation on PI substrate followed by spin coating Au nanoparticle precursor (HAuCl_4_). Re-irradiation of laser on the system brings reduction of precursor to strongly adhered Au nano particle. The electrode pattering technique can be considered as a standard method to pattern noble metals into LIG. The lowering of resistance, easy patterning of electrodes, flexibility, scalability, porosity *etc.* steers the fabrication method as efficient for flexible graphene electrodes. The Au–LIG based TENG achieved a 14.4 μW instantaneous power which was 4 times greater than the LIG based TENG. This team successfully demonstrated the compact electrode patterning technique for writing recognition. Appropriate patterning of the electrode surface is essential in tuning the properties of triboelectric nanogenerators. In general, the increased surface area of electrodes can enrich the surface charge density of the tribomaterials. Such treatment has been put forward by Zhou *et al.*^29^ who developed a Cu nanowire-based Cu mesh to maintain a strong integration with electrodes and tribomaterial. The PDMS layer on the nano-patterned mesh promotes a rough triboelectric surface in which the PDMS layer copy the patterned structure partially or fully. The mesh type electrodes provide higher mechanical strength and provide a large area of contact with the tribomaterials. Since it is a mesh design, flexibility can be ensured without compromising mechanical strength. The material innovation is sufficient to replace most of the conducting polymer-based electrodes in which mechanical strength is a challengeable question.

One of the major concerns associated with rigid materials is their low adherence to the pre-strained or pre-stretched substrates. This can be tackled by geometrically modulating the rigid material itself by making them wavy structure, wrinkled *etc.* Zhou *et al.* introduced an all in one self-powered stretchable electrode with a wavy structure by a paper-folding method.^91^ A single electrode mode TENG was fabricated with silicone rubber as the friction layer and the device encapsulating layer, whereas the carbon paper served as the flexible conductive electrode. Graphite coated sandpaper substrate was used as the highly flexible carbon paper electrode. The high surface area of the rough sandpaper substrate enhanced the loading of active graphite material which was coated by pencil drawing. Finally, the flat electrode was simply folded to obtain maximum stretchability and was encapsulated by a silicon rubber layer. The folding angle of carbon paper is found to be the deciding factor for the ultimate stretchability of this electrode. The folding angle of the carbon paper impacts the stretchability of this electrode. This TENG device was operated at 2.5 Hz under the single-electrode mode, and the test area was 3 cm × 5 cm. A *V*_oc_ of 330 V, *σ*_tr_ of 79.3 μC m^−2^ and average power density of 32 mW m^−2^ was obtained. Another major challenge associated with rigid electrode materials in wearable TENGs is the potential fracture of the materials after a certain number of working cycles. One potential way to address this concern is providing wavy configurations which can accommodate extreme strain deformations over a large number of deformation cycles. These wavy structured configurations are well explored in stretchable electrodes for electronic applications by deposition or coating of pre-stretched flexible polymeric substrates.^92–94^ Metal nanoparticles with various morphologies such as nanorods, nanoribbons are utilized in these which biaxial stretchable and highly conductive electrodes. A wrinkled PEDOT:PSS based TENG fabricated by Wen *et al.* is one of the best examples of a highly stretchable and highly conducting electrode for wearable TENGs.^3^ This transparent and stretchable TENG was fabricated by initially fixing a pre-strained poly(dimethylsiloxane) (PDMS) on a flat glass panel. Later, the pre-strained elastomeric substrate was coated with a thin film of conducting polymer PEDOT:PSS by a film is blade-coating technique. Following this, when the coated substrate was released from the glass panel, an instant formation of a wavy structure was obtained. Finally, the whole assembly was encapsulated in a PDMS substrate and Al foil served as the lead wire. This device was proved to be suitable for body motion harvesting and additionally as a human skin attachable active motion sensor. In another approach, kirigami architecture was introduced by Dong *et al.* to impart high stretchability to rigid materials.^95^ Silicon rubber was employed as the triboelectric layer. The polymer and curing agent mixture for preparing the silicon rubber substrate was poured into an acrylic plate with a network of silver-coated nylon conductive yarn. The silicon substrate was then cured to obtain the stretchable TENG device with a network of conductive yarn with a zig-zag arrangement and rhombus interlaced network. Additionally, the assembly was sealed using a flexible PDMS layer. The gradual alignment of the zigzag yarns networks, while it was stretched, provided the extension of the rhombic region. This in addition to the thickness of the PDMS layer determined the ultimate stretchability of the electrode. The TENG could be stretched to about 30% strain which is suitable for e-skin and pressure sensing applications.

### 4.2. Stretchable matrices embedded or coated with conductive materials

Flexible electrodes can be realized *via* a simple amalgamation of conductive materials such as metal particles/films, carbon nanotubes, carbon black, *etc.* with flexible substrates typically polymeric materials including PDMS, silicone rubber, polyethylene terephthalate (PET), polyimide (PI), and so on. Although thin glass and metal foil can as well be placed under substrates for flexible applications, they fail to keep electrical and mechanical integrity upon continuous deformation besides the brittleness of glass substrates when coated with thin metal films. The polymeric substrates thus find significance as feasible materials in flexible electronic applications.

**Fig. 13:**
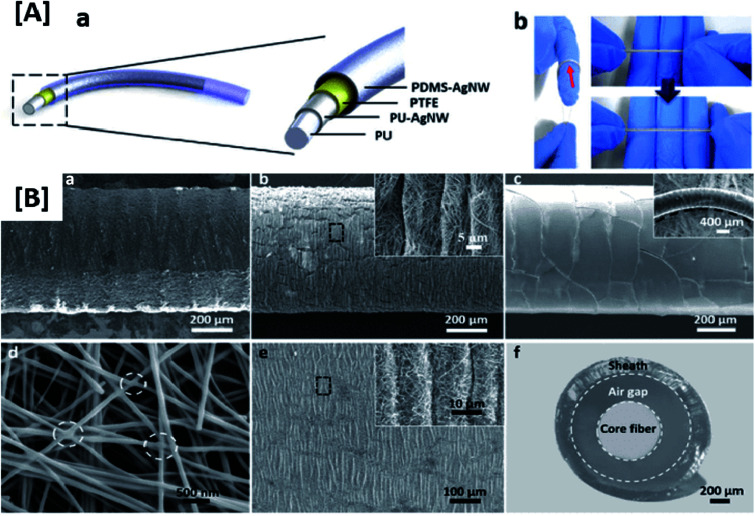
[A] (a) The schematic structural illustration of a fibre nanogenerator (FNG) without the external electric circuit (b) the photograph presenting the finger wrapped FNG device (marked by the red arrow) [B] SEM images of the (a) bare PU fibre, (b) PU–Ag NW fibre (100% pre-strained) and enlarged view (black dotted box) in the inset, (c) PU–Ag NW–PTFE core fibre and bending state of the core fibre (inset), (d) Ag NW network after annealing with nano welding junctions (inside the circles), (e) PDMS–Ag NW film and its enlarged view of the marked region (inset), (f) optical image of the cross-sectional structure of the FNG device where the air gap region between core fibre and sheath is pointed out (dotted circle) (reprinted with permission,^105^ copyright 2017 Nano Energy).

Apart from the drawbacks discussed of ITO as the flexible electrode, the high demanding processing temperature limits its combination with polymeric materials. Among various ITO alternatives, silver nanowire (Ag NWs) networks are a fascinating choice for the conducting material in a stretchable electrode on account of several hallmark factors like better conductivity, cost-efficiency, ease of manufacture, and flexible and stretchable nature.^96–98^ Unlike other plastic and organic conductive materials and buckled thin metal films, these percolating 1D nanomaterials can effectively deal with the development of strain, as well as display superior stretchability.^99,100^ In 2016, Wang *et al.* developed skin-like durable and resilient triboelectric nanogenerators which are capable of harvesting electricity under drastic mechanical conditions with a uniaxial stretchability of around 300% strain. Fig. 13 depicts the fabrication process of Ag NWs embedded silicone rubber electrodes. An acrylic sheet plated with Kapton tape is employed to drop-coat Ag NWs suspension. Over it is casted, a liquid silicone rubber followed by the removal of the Kapton strips. Then the Ag NWs–silicon rubber film can be peeled off from the acrylic sheets after proper solidification of the silicon rubber. The TENG structure can be completed by curing liquid silicon rubber over the peeled-off film by pinning copper tape as the conductive wire. This facile process of assembling a highly flexible device holds the possibility of an economic and industrially friendly production under room temperature conditions.^11^

In another case, Cheng *et al.* constructed^101^ a stretchable nanogenerator comprising a coaxial core–sheath fibre structure with a combination of core and sheath electrodes powered with Ag NWs along with a rational air gap modulation that could steer the development of microstructure variation over a range of mechanical impulses such as stretch, bend, twist, and press. Here, Ag NW ink was coated over the pre-stretched PU fibres, which, when released later, helps to form a buckling microstructure on the surface. This, in turn, improved the electromechanical stability and the surface roughness of the flexible electrode and also promoted an anchoring effect on a PTFE coating over the whole assembly.^101,102^ The sheath electrode was developed by an *in situ* polymerization and transfer method. Throughout the electrode construction, a silicon substrate was used for the development of an Ag NW percolation network which later underwent annealing treatment for the nano-welding of the Ag NW network. This consequently enhanced the electrical conductivity and structural robustness over mechanical stimulation.^103,104^ The removal of the solvent and organic residues on the Ag NW network ascribed to the thermal annealing process also promotes atomic mobility and diffusion for confined nano-welding at contact areas of neighboring nanowires. The sheath electrode developed by the attachment of the Ag NW network over the pre-stretched and released PDMS film used liquid PDMS as the binder layer. The successful transfer of the Ag NW network onto the PDMS surface was achieved through the fabrication technique employed and the PDMS–Ag NW network was formed after the curing stage (Fig. 13B). The buckled microstructures in the composite layer impacted the overall electromechanical properties of the stretchable electrode. These core and sheath electrodes were then incorporated into the device with an air gap of around 250 μm (Fig. 13).^105^

Lately, Li and co-workers developed a highly stretchable TENG for human health monitoring, wind speed sensing, and energy harvesting which employed electrospinning for the flexible electrode construction whereas a three-dimensional anti-nickel foam structure based on a porous PDMS film act as the counter triboelectric material (FSTENG).

### 4.3. Intrinsically stretching conducting electrodes

Though electrode stretchability can be obtained by altering synthesis techniques and tuning the geometric patterns, there are a few conducting materials including liquid state materials, which have extremely low Young's modulus and can be incorporated into wearable electronics. Yang and co-workers^106^ reported the use of liquid metal-based electrodes in TENGs. The liquid metal Galinstan encapsulated in silicone rubber exhibited excellent conductivity and stretchability at room temperature. Here silicone rubber acted both as encapsulation and triboelectric layer (Fig. 9). The fabricated TENG unit of area 6 × 3 cm^2^ gave a *V*_oc_ of 354.5 V, *I*_sc_ of 15.6 μA, the power density of 8.43 mW m^−2^ and transferred short circuit charge of 123.2 nC. Similarly, Shi *et al.*^42^ fabricated a TENG by encapsulating liquid PEDOT:PSS in silicone rubber. The PEDOT:PSS electrode demonstrated crack resistance up to stretching of 300% strain. The fabricated TENG gave a short circuit current of 24.9 μA, *V*_oc_ of 265 V and average power of 24.8 μW.

G. Zhao *et al.*^31^ fabricated a TENG featuring ionogel sealed in PDMS films. The unit exhibited excellent stretchability (121%) and transparency (83%) and, therefore, can be applied in wearable electronics as pressure and tactile sensor (0.39–1.46 V N^−1^). This design features a PDMS tribo electrification layer sandwiched between two ionogel films. The upper ionogel film acted as one of the friction layers and the top electrode, whereas the bottom ionogel film was the back electrode to conduct the induced charges. The ionogel films were highly stretchable and mechanically durable which rendered the device an ultimate stretch of 170 kPa at a stretch of 125% strain. Apart from that, the ionogel films exhibited an ionic conductivity of 1.9 S m^−1^ at 25 °C. A shape adaptive TENG was reported by Pu and co-workers which utilizes polyacrylamide (PAAm) hydrogel containing lithium chloride (LiCl) as electrodes (PAAm–LiCl hydrogel electrode). The synthesized hydrogel was sandwiched between commonly available elastomers *viz.*; PDMS and VHB to incorporate in a TENG giving a peak power density of 35 mW m^−2^.

In a study conducted by Li *et al.*^76^ a Cu nanowire/RGO (Cu NW/RGO) composite electrode and fluorine modified PDMS friction layer was explored. In comparison to the standard ITO electrode, Cu NWs/RGO electrode could enhance the output voltage from 28 V to 120 V, and increase the transfer charge quantity from 30 nC to 80 nC. It is also noteworthy to mention that the transmittance of the Cu NW/RGO electrode (70% in the visible spectral region) is fairly higher than that observed for a standard ITO electrode. However, the fact that the electrical properties of these two samples (which exhibited almost the same conductivity) are almost similar justifies that the major role of electrodes is to drive the current through the external circuit. It was observed that few layers of graphene were coated over Cu NWs and the Cu NW/RGO electrode exhibited a conductivity of 58.4 Ω sq^−1^ and also they were uniformly distributed to form a conductive network in the electrode. Another study conducted by Meng *et al.*^112^ developed a flexible and stretchable single electrode mode TENG device featuring the highly conductive copper metal as the electrode material and a mechanoluminescent ZnS:Cu/PDMS composite as the triboelectric layer. This multilayered coaxial TENG exhibited a thickness-dependent electrical output performance with a maximum value of open-circuit voltage (*V*_oc_) up to 21 V, short-circuit current (*I*_sc_) of 0.1 μA, and the short-circuit charge quantity (*Q*_sc_) of 8.5 nC and maximum contact area of 0.85 cm^2^. The potential difference created by the contact electrification occurring at the ZnS:Cu/PDMS and the active materials rendered the flow of electrons generating an alternating current. Moreover, the electrical output of the device was observed to be in line with the percentage stretching. This is primarily due to the increase in charge flow rate caused by the increase in contact area during the contact and separation cycles. The device was suitable for human motion monitoring and health care. Additionally, this wearable self-powered fabric TENG with an orthogonal woven structure can light up more than 60 LEDs connected in series and switch on an electronic watch by repeatedly tapping it.

Highly durable textile-based TENGs are also developed with electrodes that feature intrinsically conductive materials. This was well demonstrated in a study by Busolo *et al.*^110^ where an extremely durable core–shell structured triboelectric yarn with an approximate diameter of 850 μm was fabricated with a conductive carbon nanotube (CNT) coated with electrospun PVDF fibres. A uniform coating of the PVDF fibres was obtained over the CNT yarn using a modulated electrospinning unit. The electrospun yarns produced a *V*_oc_ and *I*_sc_ of 2.6 and 465 nA, respectively under the vertical contact separation working mode with high resistance to wear and high flexibility. Even after 20 000 stretching cycles, the yarn produced a peak power density of 20.7 μW cm^−2^ for a contact area of 0.096 cm^2^ (Table 2). The usage of an intrinsically conducting electrode with the friction layer coated over it helped to eliminate the surface patterning and chemical modifications required otherwise for better properties. Moreover, it is also noteworthy to mention that the limitations shown by those designs which feature metal overlays could be eliminated by the core–shell structure of this highly conducting and mechanically durable triboelectric yarn.

**Table 2:** Summary of various fabrication strategies used for stretchable TENG electrodes and their output performance

Electrodes	Fabrication strategy	TENG Performance	References
Ni coated polyester fabric	Electroless plating	1 μA constant current for 3 cycles	107
Ag/PVC interdigitated electrodes	Screen printing	*I*_sc_: 2.68 μA	108
		*V*_oc_:136 V	
		Power density: 38.8 mW m^−2^	
Vertically aligned Au NWs/PDMS-based triboelectric tattoo	Modified seed-mediated approach for growing Au NWs on spin-coated substrates	100–300 mV under stretching strain of 500%	109
Single crystal graphene covered Cu NW	Layer-by-layer coating	*I*_sc_: 3.6 μA	76
		*V*_oc_: 62 V	
		Max. current efficiency: 4.25 cd A^−1^ at 8760 cd m^−2^	
Graphene coated nylon fabric	Dye coating	*V*_oc_: 213.75 V	37
		*I*_sc_: 3.11	
Conductive carbon fibre cloth	Drop casting Ag loaded carbon nitride over carbon cloth	*V*_oc_: 200 V	38
		*I*_sc_: 1.1 μA	
		Power density: 3.1 μW m^−2^	
PVDF coated CNT yarn	Electrospinning	*V*_oc_ = 43.2 V	110
		*I*_sc_ = 4.25 μA	
		Power density = 11.89 μW m^−2^	
Polypyrrole deposited cotton fabric (PPy@CT)	Deposition *via in situ* polymerisation	*V*_oc_ = 200 V	111
		*I*_sc_ = 6.0 μA	
		Power density = 82 μW m^−2^	

## 5. Conclusions and future directions

Recent studies have shown great advancements in developing potential materials and fabrication techniques for TENG based wearable electronics. Intrinsically stretchable materials and soft materials in combination with rigid conductive materials have also been highly explored for wearable TENGs. Even though this review focuses more on the developments in stretchable electrodes for TENGs, it is evident that the TENG device as a whole need to be considered to address the wearability of the device. The review suggests that the development of a TENG device with only stretchable components requires more insights into potential materials and strategies. Apart from that, the complete elimination of rigid conductive materials is nearly impossible if the conductive pathways are to be retained for consistent performance. Even though the right choice of materials, fabrication strategies and geometrical patterning have helped to realize the development of stretchable TENGs to a great extent, some challenges associated with a fully wearable device still prevail. Especially, designing the electrode component with comparable flexibility to that of the triboelectric layer requires new approaches in terms of materials and methods. Also, the retention of stretchability over the entire service life of these devices with consistent output performance and minimum chances of delamination of different layers remains a challenge. This demands an adequate combination of different materials which could accommodate unpredictable deformations and extreme strain rates. Additionally, the requirements such as self-healability, transparency and water resistance should also be considered along with stretchability. Another challenge is associated with the integration of stretchable TENGs with energy storage domains: because in this case, the interface engineering between the charge generating and charge storage module is essential for the retention of charge transfer efficiency of TENGs. Few recommendations for reasonable solutions to the identified challenges are listed below:

(1) The stretchability of each component is highly important to achieve a 100% wearable TENG device. Therefore, it is essential to identify potential materials suitable for each component of the TENG assembly, which can retain the output power and cycle stability alongside flexibility.

(2) The difference in stretchability between the materials used for each component, will potentially affect the expected wearing comfort of the device. Also, this leads to an increase in the tendency for delamination between different layers within the device. Therefore, potential materials with similar (matched) mechanical properties and inherent stretchability need to be explored further.

(3) For the electrode component, more focus should be directed to exploring conducting materials that are inherently flexible and easy to integrate with the other components of a TENG. This will help to minimize the incorporation of reinforcing and conducting rigid fillers which would interfere with the stretchability of the device and other desired properties such as self-healability and transparency.

(4) New fabrication strategies, which imparts high surface area and deformability to the device needs to be explored. The recently explored geometrical patterning strategies such as wrinkled and buckled surfaces, core–shell designs, embedded structures *etc.* could be modified and extended further.

## Conflicts of interest

The authors declare no conflict of interest.

## Supplementary Material
